# Mr. Pitt's Case of Fracture

**Published:** 1805-05-01

**Authors:** Edward Pitt


### *To the Editors of the Medical and Physical Journal.*

GENTLEMEN,

ON the 26th of August, 1804, about eight o'clock in the evening, whilst the Honourable East India Company's ship Northampton, was lying at anchor in the harbour of Coringa, on the Coromandel coast, a lad about twelve years of age, with a large wound in the head, (caused by a marlin spike * falling out of the fore-top upon his head) was brought on board for assistance by the chief-mate of a country ship, they having no surgeon on board.

On clearing away the blood and shaving his head, I perceived a large wound on the right side and towards the vertex near the posterior and superior angle of the parietal bone, part of which was laid bare; it extended forward about four inches in length, parallel with the sagittal suture. As the *mental faculties* of the boy were *not at all impaired,* I did not at first conceive that any fracture had taken place, more particularly as the bone appeared perfectly smooth and without fissure when laid bare; but on lifting up the scalp and examining further, I found I was mistaken in my conjecture. I immediately dilated the wound, and discovered that the parietal bone was *fractured and depressed,* at least three inches in length, and three-quarters of an inch in breadth near the posterior and superior angle, and passing forwards nearly in the direction of the wound. I immediately applied the trephine, and raised the depressed part; no effusion had taken place; the scalp was laid down, dressed with mild digestive, and the dressings secured by a bandage and a cotton night cap.

The patient (I was informed by the officer who brought him) appeared rather convulsed as he came in the boat; but when brought on board the ship he had no symptoms of them; on the contrary, he was perfectly sensible, and answered any questions that were put to him, and continued so during the whole time of the operation. During the first night after the operation he appeared rather delirious for a short time once or twice, and had some slight febrile symptoms, and the four or five succeeding nights had a slight return of fever without delirium; after which he had no complaint except a slight head-ach now and then until the completion of the cure, Oct. 15.

When

* An iron instrument about seven or eight pounds weight.

When we read the various and contradictory symptoms enumerated by authors, of compression of the brain from external violence, and of concussion, the younger practitioner is in some measure at a loss on whom to depend for a guide, when a case comes under his own immediate care. The symptoms of concussion and compression are by most authors said to be totally distinct, while others assert there is no difference; to me the almost *only* difference appears to be, the *time* at which the alarming symptoms follow the accident.

Now in the preceding case, not one of the symptoms generally enumerated, viz. stupor, loss of sense and voluntary motion, dilated pupil, &c. were present, although the depressed part of the bone was pressing firmly on the dura mater; and had there been no external wound, it might have been difficult to have ascertained the existence of a fracture; in which case the patient would have been freely bled, had purgative medicines given him, and he would have been supposed to be doing well, until, in a short time, all the alarming symptoms would have shown themselves; violent inflammation would in all probability have taken place in the membranes of the brain, which might have terminated fatally, more particularly when the nature of the climate is considered, where, from the excessive heat, determination to the head is easily induced; and when once this action takes place, is not so easily removed as in our own climate.

Perhaps it may be deemed unwarrantable by some to have applied the trephine so soon, without the existence of any alarming symptoms; but I would ask, if it was not to be presumed they would shortly have taken place, if the piece of bone causing the compression had not been elevated? It is well known the existence of a fissure is not of itself a sufficient reason for trepanning, unless attended with alarming symptoms indicating extravasation or effusion; but when, as in this case, added to the fracture, a considerable portion of the skull is depressed, very few I imagine, would hesitate as to the means to be pursued for the relief of the patient. Yet I do not mean to say, I would apply the trephine in all cases of fracture to prevent inflammation taking place; that were I think unwarrantable, although advised by Mr. Pott; but I would ask how the removal of a piece of skull (unless pressing on the brain) can prevent inflammation? it would rather accelerate than retard that action.

I have related the preceding case merely to prove that we are not always to look for particular symptoms in these cases, cases, and consequently that alarming fractures with compression may take place, and not be characterized by the particular symptoms generally enumerated.

I am, &c.

*March* 13, 1805. EDWARD PITT.

